# Prevention, prevention and prevention

**DOI:** 10.1186/s13584-019-0310-z

**Published:** 2019-05-02

**Authors:** Shlomo Paul Zusman

**Affiliations:** Division of Dental Health, Ministry of Health, Jerusalem, Israel

**Keywords:** Dental health, Dental care, Prevention of dental disease, Dental treatment needs

## Abstract

A recent study by Levy et al. presents the dental treatment needs of a large sample of combat soldiers. They found that 80% need some dental care.

It is unfortunate that so many, educated, otherwise healthy young adults are in need of dental care and it indicates that more prevention of dental disease is needed.

In last years, the Ministry of Health started two projects to prevent dental disease among the very young: In Mother and Child Health Centers and in kindergartens. In a couple of years, these projects will be widely implemented. With the School Dental Service, these two projects will cover all ages from birth to 18.

In the future, with these programs implemented, soldiers should need less dental treatment.

## Main text

The recent IJHPR article “The association between caries related treatment needs and socio-demographic variables among young Israeli adults: a record based cross sectional study,” by Levy et al. [1], presents the dental situation and dental treatment needs of a large sample of 13,398 young adults (soldiers). The sample represented an educated group of healthy, young adults.

According to the findings of the examinations, their dental treatment needs are higher than we would expect. At the age of 18–19, only 18% needed no treatment, whereas 82% needed treatment. “The mean number of teeth in need of restorations was 1.96 ± 2.59 per soldier (range 0–24), 0.07 ± 0.44 for root canal treatments (range 0–12), and 0.05 ± 0.28 for extractions (range 0–5)”. Prior to 2010, dental treatment for children and adolescents was the sole responsibility of their parents and left to their means to pay for it in a largely privately funded delivery system.

In 2010, new legislation was passed reforming dental care for children. Dental care for children was added to the basket of services of the National Health Insurance Law. Coverage of children and young adults was broadened gradually and since January 1, 2019, it now extends from birth to age 18. Most recently, additional legislation has extended coverage for both restorative and prosthetic to the elderly [2]. This is an important change that can solve the problem of untreated disease, but it would be better to keep the teeth healthy in the first place.

No doubt, the best way to tackle dental disease and disability is by prevention. The earlier the prevention is started, the better.

Community water fluoridation (CWF) is by far the best way to prevent dental caries at community level [3]. It was the main policy of the Ministry of Health for many years with unanimous, unshaken support of the Ministry’s professional staff. Israel has a very diverse population, and the diversity is reflected in its democracy and the multiple parties represented in its parliamentary government coalitions. CWF was stopped in Israel in 2014 because the Minister at that time had a different set of priorities. There have been elections since then; the current Minister supports CWF; but despite all the efforts of the Ministry, it has not yet been restarted. We already are seeing the result of halting this important public health measure with dental health of children deteriorating [4]. We hope it will be re-started soon.

The Ministry of Health’s activity does not stop with the reform of dental health coverage for the population. The Ministry acts in the prevention arena, as well. Lately, prevention of dental disease has been extended to Mother and Child Health Centers [5], where public health nurses perform Caries Risk Assessment and apply the appropriate preventive measures. This initiative started a couple of years ago in the south of the country, and it is spreading gradually to other areas as well. Funded by the Ministry of Health, it will be universal in a few years.

In kindergartens, tooth brushing is done by the 3–4 year old children, supervised by the teachers [6]. Each school-year, more and more kindergartens join. The model was adapted to special education kindergartens too [7]. This project also is funded by the Ministry of Health, and it will be universal in a few years.

These two programs complement the School Dental Service, wholly funded by the Ministry of Health since 2010. It provides outreach preventive services to all children in the compulsory education, from preschool to 10th grade.

It is worth mentioning that preventive measures for children are free of co-payment in the basket of services of the National Health Insurance Law. Those services are provided to children that show up at the dental clinics, i.e. they are dependent on the parents’ awareness, hence not universal. For this reason, the outreach programs mentioned earlier that are universal and cater to all children regardless of parents’ awareness are an important addition to the national effort to prevent dental disease.

## Conclusions

Only prevention in early childhood can see to it that young soldiers will be drafted with substantially less dental treatment needs. We expect that the effort invested in these national programs will be effective in achieving this result.

## Abbreviations

MoH: Ministry of Health
RCT: Root Canal Treatment
